# Self-inflicted partial epiphyseolysis of the distal femur—a case report

**DOI:** 10.3389/fped.2024.1425358

**Published:** 2025-01-23

**Authors:** Anna Kanewska, Johannes Krause, Mario Perl

**Affiliations:** Department of Orthopaedic and Trauma Surgery, University Hospital Erlangen, Friedrich-Alexander-Universität Erlangen-Nürnberg, Erlangen, Germany

**Keywords:** epiphyseal fractures, epiphyseal fracture of the distal femur, spina bifida, case report, Kirschner wire (K-wire)

## Abstract

**Introduction:**

Injuries to the epiphyseal plate are of great concern as they can affect bone growth. Although epiphyseal fractures are common in adolescents, fractures of the distal femoral epiphyseal plate are rare.

**Case presentation:**

We present a case of a Salter–Harris type 1 fracture of the distal epiphysis of the femur that was self-inflicted by a patient with paraplegia due to spina bifida. The patient was brought to the pediatrician's attention during a routine checkup with an apparent swelling of the right thigh. Upon presentation, we performed a radiograph and an additional MRI, which revealed a partial ventero-medial epiphyseolysis, consistent with a Salter–Harris type 1 fracture. Due to the dislocation, we indicated closed reduction with K-wires. Repositioning was performed using a modified Kapandji maneuver and was completed with additional K-wires.

**Conclusion:**

Distal epiphyseolysis is a relatively rare injury that can lead to serious complications. Therefore, although rare, epiphyseal fractures should be considered in pediatric patients presenting with uncertain limb swelling.

## Introduction

In growing bones, the border between the initial cartilage ossification and the newly forming cartilage is called the epiphyseal plate. It is responsible for longitudinal bony growth, while the periosteum is for thickness growth (1, 2). The epiphyseal plate is organized into several zones, namely, the quiescent, proliferative, prehypertrophic, and hypertrophic zones. It exhibits high remodeling activity and a higher cellular density compared to diaphyseal bone (3). It is therefore more susceptible to shear and bending forces and is considered a “locus minoris resistentiae” (4).

Therefore, epiphyseal fractures are quite common in adolescents aged 10–15 years, especially among boys, accounting for up to 60% of all fractures (4). Yet, distal femur involvement is rare and comprises less than 5% of epiphyseal injuries (5) and less than 1% of pediatric fractures (6). Typical trauma mechanisms are hyperextension and torsional forces around the knee, often combined with high impact (7). In general, treatment of epiphyseal fractures depends on several factors, including the bone involved, location within the bone, fracture crossing the epiphysis, age and gender of the patient, degree of dislocation, socioeconomic status of the patient, patient and caregiver preference, risk expectancy, and prevailing risk factors (3). If not treated adequately, fracture healing is prone to growing disturbances due to the formation of bone bridges during the healing process. The direction of growth deviation depends on the localization of the bone bridge (3). Despite their rarity, injuries of the distal femoral epiphysis are more likely to be associated with complications compared to injuries of the upper extremity (8, 9).

To date, there are no standardized clinical trials for the treatment of distal femoral epiphyseal fractures. We present a case of a Salter–Harris type 1 fracture of the distal femoral epiphysis that was self-inflicted by a patient with paraplegia due to spina bifida. Not only is this a rare fracture entity in itself, but it also has an unusual fracture mechanism and circumstances. As mentioned above, distal femoral epiphyseal fractures are prone to complications, especially given the patient's individual risk factors with an increased likelihood of delayed diagnosis or improper care, thus heightening the risk for complications such as nonunion, malunion, or growth disturbances.

The purpose of this case presentation is to emphasize the importance of prompt recognition in the management of epiphyseal fractures and the need for standardized clinical trials for their treatment.

## Case report

### Patient information and clinical findings

The patient is an 11-year-old Caucasian boy with partial paraparesis due to spina bifida, meaning that active movement of the lower limbs has not been possible since birth. His pediatrician noticed swelling in his right thigh during a routine checkup.

Since he was paraplegic, a history of pain in his lower limbs was not possible. Upon further examination, it was found that, “out of boredom,” he had been pulling and tilting his right tibia for a few days, as well as rotating it sideways along the knee axis.

### Diagnostic assessment

**Figure 1:**
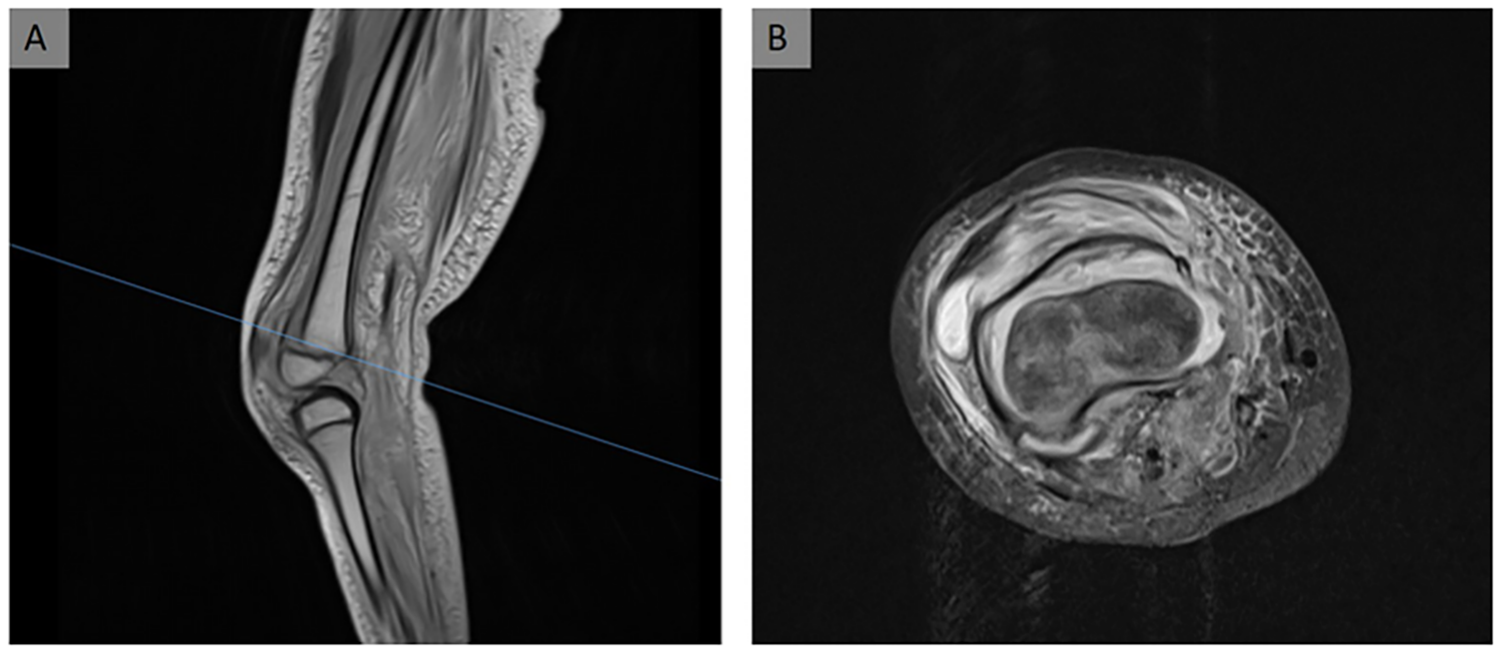
MRI scan of the patient's right knee. (**A**) Epiphyseolysis of the distal femur in the sagittal view. Because the epiphyseal scar is not crossed and the fracture line is along the epiphyseal scar, this is classified as a Salter–Harris type 1 fracture. (**B**) Axial view of the right knee. The femoral condyle appears to be dislocated ventromedially from the femoral metaphysis.

Given the history of shearing forces applied to the right knee, including the distal femoral epiphysis, we performed a right knee radiograph for initial evaluation. A magnetic resonance imaging (MRI) scan was performed for further evaluation and to rule out any inflammatory processes (Figure 1).

Imaging revealed a Salter–Harris type 1 fracture of the distal femur, characterized by rotational displacement and the presence of a hematoma beneath the torn periosteum.

Due to the dislocation of the distal femoral epiphysis with a high risk of growth disturbances (8) and the underlying paraparesis of the patient, we placed the indication for surgical repositioning. The aim was to establish a load-stable reposition.

### Surgical technique

After surgical disclosure and obtaining written consent from the legal guardian and the patient, the patient was intubated and placed in the supine position. The right femoral condyle was manually aligned under axial traction and radiographic control. This slightly improved the medioventral dislocation.

Reduction of pediatric fractures should be attempted using a closed reduction before considering an open reduction (3). However, there is a higher risk of growth retardation with multiple repositioning maneuvers (10). A possible percutaneous repositioning technique is the Kapandji technique. Originally used in repositioning fractures of the radial epiphysis, this technique has been adapted for use in other types of injuries (11). The Kirschner wire (K-wire), called the Kapandji wire, is inserted into the fracture-split and used as a hypomochlion to lever the distal fragment into the correct position under radiographic control. After achieving the desired position, it can then either be removed or positioned slightly further to act as an osteosynthesis device.

In this case, we decided on a Kapandji maneuver via a medioventral approach because the distal fragment presented as one block (Figure 1), was still rotated medioventrally after manual repositioning, and was well accessible for percutaneous K-wire positioning. Therefore, a K-wire was inserted into the fracture-split by blunt preparation and used as a hypomochlion to reposition the distal femoral fragment. We started with the medial condyle since the epiphysis was rotated medioventrally.

For osteosynthesis in fractures in the metaphyseal square, the widely accepted technique is K-wire osteosynthesis, forming a planar cross (3, 4, 9, 12). It is important to not cross the epiphyseal plate more often than necessary to avoid the formation of bone bridges (12, 13), which can lead to growth disturbances.

Hence, another K-wire was inserted to secure the repositioned fragment in that position, creating a planar cross. We ensured the 2.2-mm K-wires crossed proximal to the fracture site to prevent rotational instability (5).

**Figure 2:**
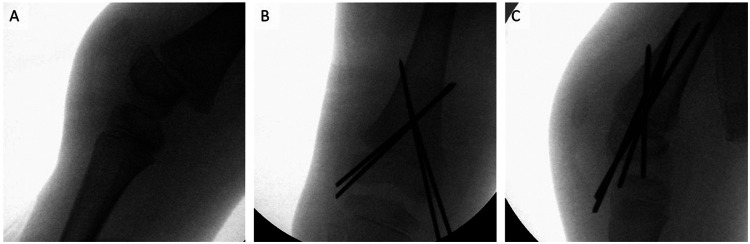
Intraoperative radiograph of the right knee with K-wires inserted. (**A**) Sagittal view of the intraoperative radiograph. The tibia is being held in traction. (**B**) Coronal view after the insertion of four 2.2-mm K-wires. (**C**) Sagittal view. The epiphysis is correctly aligned axially.

To further stabilize the relatively high-volume femoral metaphysis, two additional K-wires were inserted parallel to the two already in place (Figure 2). After confirming stable joint placement, we assessed the swelling on the right thigh. Since there was no evidence of restraint, we decided not to surgically open the swelling. An attempt to puncture the hematoma was made but was unsuccessful.

A cotton wool bandage was applied to the affected area, and immobilization was performed using a thigh splint. The removal of the K-wires was scheduled for 3–4 months after surgery, depending on bony consolidation.

### Follow-up

As epiphyseal fractures of the femur carry a higher risk of growth discrepancies due to epiphyseal arrest (6, 8), functional controls are advised every 3 weeks, accompanied by radiographic assessments after surgery (intraoperative) and during the consolidation phase (3).

**Figure 3:**
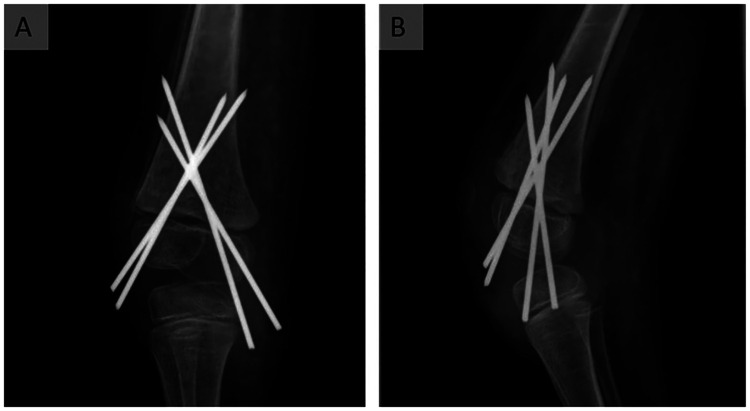
Follow-up radiograph of the right knee, 1 month after surgery. The radiograph shows a correct axial alignment of the epiphysis, 1 month after surgery of the Salter–Harris type 1 fracture in coronal (**A**) and sagittal (**B**) views. There are no signs of secondary dislocation or wandering of the K-wires. Mild osseous consolidation can be seen in (**B**).

Immobilization was achieved using a femoral soft cast. The patient showed limited compliance in addition to the self-inflicted trauma mechanism. Therefore, we ordered a radiographic follow-up 1 month after surgery. It confirmed maintained correct axial alignment of the fracture with no evidence of secondary dislocation (Figure 3). However, in the controls, the skin over the medial femoral condyle showed increasing signs of dehiscence, finally leading to perforation by a K-wire 1 month after surgery. Given the early signs of consolidation and correct fracture alignment, the perforating K-wire was removed. Immobilization was maintained in a soft femoral cast until the remaining K-wires were removed 3 months postoperatively.

The patient received physical therapy twice a week and was mobilized using a reciprocal walking orthosis. No further complications related to the fracture were observed until the 1-year follow-up.

## Discussion

Despite its rarity, a similar case of epiphyseal fracture of the distal femur caused by multiple manipulations rather than a traumatic accident was described by Friedman and Blevins (14). It deals with a posterior distal femoral epiphysis in an adolescent caused by multiple tender manipulations of the knee joint under analgesic circumstances. However, this was an iatrogenic manipulation that occurred during a follow-up surgery for a proximal tibial fracture to extend the impaired postsurgical motion range. In our literature review, we could not detect a similar case of a self-inflicted fracture due to manipulation by the patient.

Epiphyseal injuries are quite common in adolescents (5, 9), with a higher prevalence observed in boys. This gender preference could be explained by the later closure of the epiphyseal plate in males compared to females (7). There are distinct types of epiphyseal injuries, depending on their underlying mechanisms. These injuries can be categorized as injuries due to overuse, growth disturbance, and trauma (4). An example of an atraumatic epiphyseal injury due to growth disturbance is the epiphyseolysis capitis femoris, which is quite common in obese male adolescents (15). Multiple microtraumas or systemically caused infarcts can lead to aseptic bone necrosis and growth disturbance (4).

In our case, the epiphyseolysis was due to trauma, even though the trauma mechanism is unusual. In the epiphyseal plate, more precisely up to 60% in the hypertrophic zone (16), longitudinal growth occurs. In the transition zone, the newly formed cartilage transitions into bone substance. The closer to the metaphysis, the higher the cellularity relative to the matrix, making it more vulnerable to shear forces (3). Furthermore, the frame-building collagenous fibers run horizontally parallel and cross only when passing the construction zone, eventually forming a grit. Therefore, the epiphyseal construction zone is vulnerable to compression, traction, or shearing and is regarded as a “locus minoris resistentiae” (5).

Epiphyseal fractures most commonly occur in the upper extremity (3, 4, 9). Our patient's epiphyseolysis of the distal femur seems to be very rare, accounting for only 0.3% of distal femur injuries (5) and less than 1% of pediatric fractures (6). Transition fractures of the distal femur are more common than epiphyseolysis (3). These fractures peak around age 12, slightly older than our patient. Back when horse-drawn wagons were the primary means of transport, distal femoral epiphyseolysis was more common, as adolescents were at risk of sustaining such injuries by catching their leg in hyperextension while trying to get on the vehicle (14).

As mentioned in the Introduction section, there are criteria for deciding upon a therapy. Various classification systems serve as guides for assessing severity, of which several exist for pediatric fractures. The LiLa classification is recommended by Lutz von Laer. In addition to the AO classification, which can be used for adult fractures, it includes the degree of dislocation (3). The most common classification was established by Salter and Harris, which describes the fracture course in comparison with the epiphyseal plate (17). To be mentioned, the less-known Ogden classification differentiates the injuries not only by localization but also by causes. For instance, patients with systemic diseases like myeloproliferative diseases or spina bifida, as existing in our patient, fall into its own category “1A” (18).

It is quite crucial whether the fracture course crosses the epiphysis or not. The outcome of epiphyseal injuries also depends on the morphology and correlates with the Salter–Harris classification (8). Exposure to torsion and shearing can separate the horizontally parallel collagenous fibers in the epiphyseal scar, resulting in a Salter–Harris type 1 injury (5), as seen in our case.

When assessing the fracture, it is important to achieve timely repositioning. If the fracture is treated inadequately, bone bridges might form, which obstruct growth. Studies have shown that this process occurs soon after a trauma (16), which is why early repositioning and, in case of a broad fracture gap, compressive osteosynthesis (3) are important. Specifically, rotatory displacements should be corrected since remodeling can occur only in the direction of movement of the peculiar joint (3). In our case, the direction of movement would be knee extension and knee flexion. The fracture fragment exhibited a medioventral rotatory displacement, which made surgical intervention necessary.

Moreover, distal femur epiphyseolysis, in particular, is more likely to cause epiphyseal growth arrest, making early reposition crucial (3) in our case. In general, growth retardation seems to occur up to six times more often in injuries to the lower extremities compared to the upper extremities (9). One plausible reason might be that blood-supplying vessels of the femur are more likely to cross over the bone prior to entering, unlike any other bone where the vessels usually enter orthogonally (4).

Bone repositioning itself can mimic the effects of a new fracture. Studies have shown that the risk of growth retardation significantly increases with repeated repositioning maneuvers. Specifically, by the third repositioning, the risk can rise up to five times, i.e., in half of the fractures, compared to the initial maneuver (10).

Therefore, repositioning attempts should impact the epiphyseal scar as little as possible. When comparing reduction by crossed Steinmann pins, cannulated screws, open reduction, and external fixation, complications are up to twice as likely when the epiphysis is not spared (8). In the literature, crossed Steinmann pins are the most common method for repositioning distal femur epiphyseolysis (3, 4, 9), although a case of closed reduction using an Ilizarov fixator has also been reported (19).

Experimental studies on wires crossing the epiphyseal scar have shown the formation of ossification bridges in the drill channel since these wires are thin enough to permit the epiphysis to glide past them during the growth process. These bridges discontinue horizontally, enabling distraction in the growth direction. This prevents osseous consolidation and allows further longitudinal growth (12). This was only observed in K-wire osteosynthesis, while screw osteosynthesis is more likely to cause bone bridges.

Because of the location of the fracture and the impaired pain sensitivity of our patient, there was a higher risk for complications like secondary dislocation or growth retardation. Therefore, we conducted an early radiographic checkup, which did not show signs of complications. However, the patient suffered from a skin perforation on the medial femoral condyles by one of the K-wires. Although secondary skin perforation is a possible risk of K-wire osteosynthesis, this risk was heightened in our case due to the lowered pain sensibility and compliance of the patient. This emphasizes the importance of regular clinical follow-ups and an early reposition of epiphyseal fractures.

## Conclusion

Especially when pain sensation is restrained, epiphyseal injuries should be excluded upon subtle signs like swelling or a corresponding anamnesis. Prompt repositioning of epiphyseal fractures is important to prevent growth disturbances.

## Data Availability

The original contributions presented in the study are included in the article/Supplementary Material; further inquiries can be directed to the corresponding author.
